# Clinical genomic profiling to identify actionable alterations for very early relapsed triple-negative breast cancer patients in the Chinese population

**DOI:** 10.1080/07853890.2021.1966086

**Published:** 2021-08-16

**Authors:** Liye Wang, Qinglian Zhai, Qianyi Lu, Kaping Lee, Qiufan Zheng, Ruoxi Hong, Shusen Wang

**Affiliations:** Department of Medical Oncology, Sun Yat-Sen University Cancer Center, State Key Laboratory of Oncology in South China, Collaborative Innovation Center for Cancer Medicine, Guangzhou, PR China

**Keywords:** Triple-negative breast cancer, next generation sequencing, clinical relevant genomic alterations, mutation, pathway, Chinese patients

## Abstract

**Background:**

Triple-negative breast cancer (TNBC) represents about 19% of all breast cancer cases in the Chinese population. Lack of targeted therapy contributes to the poorer outcomes compared with other breast cancer subtypes. Comprehensive genomic profiling helps to explore the clinically relevant genomic alterations (CRGAs) and potential therapeutic targets in very-early-relapsed TNBC patients.

**Methods:**

Formalin-fixed paraffin-embedded (FFPE) tumour tissue specimens from 23 patients with very-early-relapsed TNBC and 13 patients with disease-free survival (DFS) more than 36 months were tested by FoundationOne CDx (F1CDx) in 324 genes and select gene rearrangements, along with genomic signatures including microsatellite instability (MSI) and tumour mutational burden (TMB).

**Results:**

In total, 137 CRGAs were detected in the 23 very-early-relapsed TNBC patients, averaging six alterations per sample. The mean TMB was 4 Muts/Mb, which was higher than that in non-recurrence patients, and is statistically significant. The top-ranked altered genes were TP53 (83%), PTEN (35%), RB1 (30%), PIK3CA (26%) and BRCA1 (22%). RB1 mutation carriers had shorter DFS. Notably, 100% of these patients had at least one CRGA, and 87% of patients had at least one actionable alteration. In pathway analysis, patients who carried a mutation in the cell cycle pathway were more likely to experience very early recurrence. Strikingly, we detected one patient with ERBB2 amplification and one patient with ERBB2 exon20 insertion, both of which were missed by immunohistochemistry (IHC). We also detected novel alterations of ROS1–EPHA7 fusion for the first time, which has not been reported in breast cancer before.

**Conclusions:**

The comprehensive genomic profiling can identify novel treatment targets and address the limited options in TNBC patients. Therefore, incorporating F1CDx into TNBC may shed light on novel therapeutic opportunities for these very-early-relapsed TNBC patients.

## Introduction

Breast cancer is the most frequently diagnosed cancer and results in the second most common cancer mortality among the Chinese female population [1]. Abundant evidence suggests that breast cancer has clinical and molecular heterogeneity. Triple-negative breast cancer (TNBC) is immunohistochemically characterized by a lack of human epidermal growth factor receptor 2 (also defined by a lack of HER2 amplification by FISH), and oestrogen receptor and progesterone receptors expression [2]. TNBC is regarded as the most aggressive breast malignancy and accounts for approximately 19% of all breast cancers in the Chinese population [3]. Compared with other types of breast cancer, TNBCs have higher histologic grades and a higher proportion of lymph node metastases (cN status) at presentation, which contributes to worse disease-free survival (DFS) and overall survival (OS) [4]. Due to the lack of specific targets for therapy, TNBC represents a particular treatment challenge. Once diagnosed with metastatic TNBC, despite optimal systemic chemotherapy, few patients survive longer than 5 years [5]. Patients with early TNBC experience the peak risk of recurrence within 3 years of diagnosis [6]. Due to the heterogeneity of TNBC, personalized treatment strategies based on detecting and targeting tumour-specific alterations would be an effective treatment choice for the 60–70% of patients with TNBC who do not fully respond to chemotherapy or whose tumour progresses after chemotherapy [7].

Faced with these challenges, next-generation sequencing (NGS) provides us with molecular profiles of tumours from individual patients for the direction of treatment. NGS has increased the identification of previously unrecognized genes that may also be associated with improved therapeutic response and development of resistance to therapies. The feasibility of genomic mutation/alteration testing as a guide to treatment has been demonstrated by a multicentre, prospective trial (SAFIR01/UNICANCER) [8]. Recently, comprehensive genomic profiling (CGP) using the hybrid capture-based NGS was performed on all types of breast cancer and revealed the feasibility for finding therapy targets in patients with relapsed and refractory disease [9]. Given the inherently aggressive biological behaviour of TNBC, it is reasonable to perform genetic testing on patients, especially for relapsed patients, and make treatment decisions based on genetic testing results.

Moreover, CGP can reveal specific genomic alterations (GAs) associated with the biological behaviour of cancer, such as the early relapse of TNBC. In the current study, we defined tumour recurrence within 24 months as very-early-relapsed TNBC. We compared the genomic features of TNBC patients who had long DFS with those of very-early-relapsed TNBC, aiming to identify predictive genomic factors in very-early-relapsed breast cancer patients. The other objective of the current study was to reveal novel treatment targets and provide clinicians with targeted therapeutic options in very-early-relapsed TNBC patients.

## Materials and methods

### Patient inclusion and tissue sample acquisition

This was a retrospective study of TNBC patients with cancer treated at Sun Yat-Sen University Cancer Center. Formalin-fixed paraffin-embedded (FFPE) biopsy specimens from 36 TNBC patients, including 23 very-early-relapsed TNBC patients and 13 no-recurrence TNBC patients were obtained with the approval of the Sun Yat-set University Cancer Center (SYSUCC) Institutional Review Board. Biopsies were collected between 2012 and 2018 with consideration to the quality of FFPE specimens. Inclusion criteria were patients histologically confirmed ER-negative (less than 1%), PR-negative (less than 1%) and HER2 non-over expressing by immunohistochemistry (IHC) (0, 1) or non-amplified by fluorescence *in situ* hybridization (FISH). All TNBC patients never received chemotherapy and radiation therapy before materials were collected. Baseline demographics and survival data were extracted from the clinical record.

### Clinical review

Patient medical records were assessed for demographics, pathological features, adjuvant therapy received, time of recurrence and outcomes, which were measured DFS time as defined by the time from the surgery of primary breast cancer until the diagnosis of tumour relapse.

### Genetic alteration assessment

FFPE tumour tissue specimens from 23 very-early-relapsed TNBC patients were tested by FoundationOne CDx (F1CDx). F1CDx is a CGP platform that applies NGS to *in vitro* diagnostics with a hybrid capture-based target enrichment approach and whole-genome shotgun library construction. The F1CDx-targeted NGS platform has been described and validated before, and the methods are briefly described here [10]. F1CDx is performed exclusively as a laboratory service using DNA extracted from FFPE tumour samples. The assay employed a single DNA extraction method from routine FFPE biopsy or surgical resection specimens, 50–1000 ng of which underwent a whole-genome shotgun library construction and hybridization-based capture of all coding exons from 309 cancer-related genes, one promoter region, one non-coding (ncRNA) and select intronic regions from 34 commonly rearranged genes, 21 of which also included the coding exons (Supplementary Tables 1 and 2). In total, the assay detected alterations in a total of 324 genes. Using the Illumina^®^ HiSeq 4000 platform, hybrid capture-selected libraries were sequenced to high uniform depth (targeting > 500× median coverage with >99% of exons at coverage >100×). Sequence data were then processed using a customized analysis pipeline designed to detect all classes of GAs, including base substitutions, indels, copy number alterations including amplification and homozygous gene deletions, and selected genomic rearrangements such as gene fusions. Additionally, genomic signatures including microsatellite instability (MSI) and tumour mutational burden (TMB), were reported.

### Statistical analysis

Statistical analysis of all genes was based on a dichotomy (i.e. presence/absence of any alteration). Differences in alteration frequency and TMB between groups were determined using Chi-square and Fisher’s exact test. Statistical significance was defined as a *p* value less than .05.

### Immunohistochemistry

Standard 5-μm paraffin-embedded tissue sections from patient no. 648 were stained using an anti-ROS1 rabbit monoclonal antibody (Abcam, Cambridge, UK; clone EPMGHR2) applied at different dilutions (usually from 1:100 to 1:250).

### *Fluorescence* in situ *hybridization*

ROS1–EPHA7 fusion was determined by FISH testing on a 4 μm FFPE tissue specimens from patient no. 648.

Rearrangements of ROS1 (6q22) and EPHA7 (6q16) were independently detected using a laboratory-developed dual-colour break-apart probe (BAP) strategy probe set. 5′ and 3′ of probes of ROS1 and EPHA7 were labelled with red and green fluorescence bacterial artificial chromosome (BAC), respectively. BAC clone probes flanking the target genes were obtained from Invitrogen (Waltham, MA). DNA from each BAC probe was labelled with fluorochromes by nick translation. FFPE sections were deparaffinized, pre-treated and then hybridized with the denatured probes. Following overnight incubation, the slides were rinsed, stained with 4′,6-diamidino-2-phenylindole (DAPI), mounted and analysed using a Nikon fluorescence microscope (Nikon ECLIPSE 80i, Tokyo, Japan).

## Results

### Cohort

A total of 36 FFPE TNBC surgery samples were obtained at the Sun Yat-sen University Cancer Center between 2013 and 2018 from 23-very-early-relapsed patients and 13 patients who did not relapse for more than 3 years after surgery.

All the 23 very-early-relapsed patients in our study suffered disease recurrence within 2 years after surgery, and the average DFS was 11 months (3–23 months). All the patients were females and their median age was 50.65 years (27–67 years); 87% (*n* = 20) of TNBCs in our study received modified radical mastectomy, and the remaining patients received a partial mastectomy. At the time of diagnosis, about 52% (*n* = 12) of the TNBC patients were at an early clinical stage (stage I or stage II), and 48% (*n* = 11) of patients were at an advanced stage (stage III). Except for one patient diagnosed as invasive lobular carcinoma, the other patients were invasive ductal carcinoma. Nearly, half of the patients (*n* = 11) had visceral metastases after disease recurrence, the other patients had local recurrence and/or lymph node metastases (Table 1).

**Table 1.** Clinicopathologic characteristics of tested individuals.

Variable	Early recurrence *N* = 23	No recurrence *N* = 13	*p* Value
Mean age at diagnosis	50.65	46.62	.250
Lymph node state			
Positive	18 (78.2%)	6 (46.1%)	.071
Negative	5 (21.7%)	7 (53.9%)	
Mean tumour size			
T1	5 (21.7%)	8 (34.8%)	.05
T2	16 (69.6%)	5 (21.7%)	
T3	2 (8.7%)	0	
Stage			.003
I–II	12 (52.2%)	13 (100.0%)	
III	11 (47.8%)	0	
Historical grade			
I	0	0	.547
II	3 (13.0%)	2 (8.7%)	
III	18 (78.3%)	11 (47.8%)	
Missing	2 (8.7%)	0	
Type of surgery			
Lumpectomy	3 (13.0%)	2 (15.4%)	.605
Mastectomy	20 (87.0%)	11 (84.6%)	
Chemotherapy treatment			
Positive	19 (82.6%)	13 (56.5%)	.280
Negative	1 (4.3%)	0	
Missing	3 (13.0%)	0	

For the 13 TNBC patients who did not relapse for more than three years after surgery, the average DFS was 51.8 months (37–83 months). At the time of diagnosis, all patients were at an early clinical stage (stage I or stage II). There was statistical difference in stage between two groups (*p*= .003) (Table 1).

### Mutation prevalence

All the 36 FFPE samples were subjected to comprehensive genomic profiling. A total of 137 GAs were identified in the 23 very-early-relapsed TNBCs, with an average of 5.9 GAs per patient. Among the 13 non-relapsed TNBC patients, we detected 54 Gas in total, and the average number of GAs was 4.1. There was no statistical difference between the two groups.

**Figure 1.**
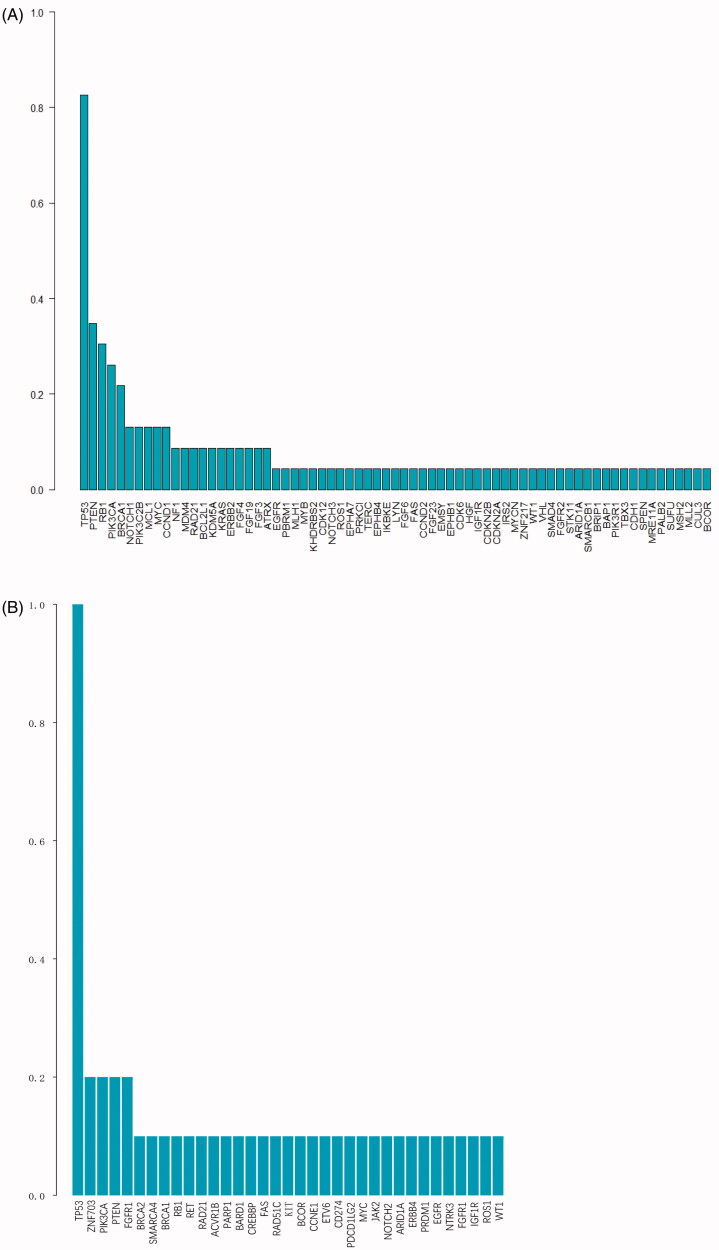
The frequency of the genomic alterations in triple-negative breast cancer (TNBC).

The effect of age (*p*= .233) and tumour stages (*p*= .639) on GAs was not statistical difference. The frequency of the GAs in a very-early-relapsed TNBC group is shown in Figure 1(A). The most frequently altered genes were TP53 (83%), PTEN (35%), RB1 (30%), PIK3CA (26%), BRCA1 (22%), NOTCH1 (13%), MYC (13%) and CCND1 (13%) in the very-early-relapsed TNBCs (Figure 1(A)). Seven patients with RB1 mutations had shorter DFS than patients without RB1 mutation, which was statistically significant (HR = 0.303, *p*= .014). The 137 GAs observed included 38 base substitutions (27.7%), 28 short insertions/deletions (20.4%), 46 focal amplifications (33.5%), nine losses (6.6%) and 16 rearrangements (11.7%). In the recurrence-free group, the most frequent mutations were of TP53 (100%), ZNF703 (23.1%), PIKCA (15.4%) and PTEN (15.4%), with the other mutations each being detected in only one patient (Figure 1(B)). Although RB1-mutated patients had shorter DFS in the very-early-relapse group, no difference was found in the RB1 mutation frequency between the two groups. Type of alterations in the recurrence-free group were different compared with the very-early-relapsed TNBCs, including 20 base substitutions (37.0%), six short insertions/deletions (11.1%), 22 focal amplifications (40.7%), four losses (7.4%) and two rearrangements (3.7%). The percentage of short insertions/deletions and rearrangements was higher in the very-early-relapsed TNBC group but substitutions showed the opposite trend.

**Figure 2.**
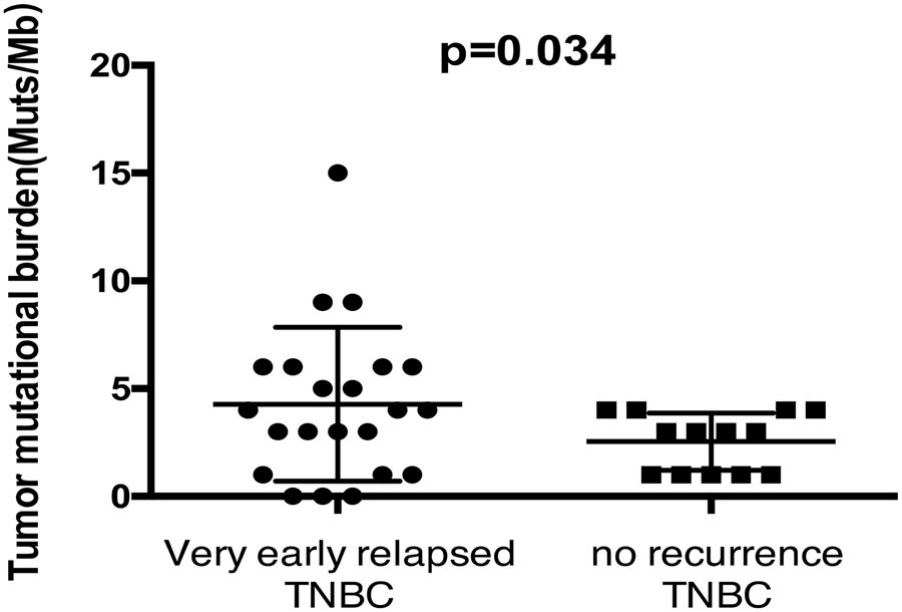
Tumour mutational burden in two groups.

Compared with 2.54 Muts/Mb in the non-recurrence group, the mean TMB of the very-early-relapsed TNBCs was 4 Muts/Mb, ranging from 0 to 15 Muts/Mb, and the difference was statistically significant (Figure 2). Expect for only one patient who had MSI-intermediate tumour, all patients harboured microsatellite stability (MSS) tumours. When comparing stage I–II recurrence-free patients with stage I–II very-early-relapsed patients, no statistical difference was found in TMB (*p*= .532).

### Clinically relevant genomic alterations (CRGAs) and potential therapeutic targets

In the very-early-relapsed TNBC group, all the patients had at least one CRGA, which is defined as a GA linked to drugs on the market or under evaluation in mechanism-driven clinical trials. Treatment recommendations based on GAs were suggested for 87% (20/23) of the patients according to at least one actionable alteration. The most frequently observed actionable alterations that were observed in the most frequently actionable targets included the following: PTEN (34.7%, *n* = 8), PIK3CA (26.1%, *n* = 6), BRCA1 (21.7%, *n* = 5) and CCND1 (13.0%, *n* = 3). We also detected 2 KRAS amplification, an ERBB2 and ERBB2 P780_Y781insGSP amplification, two NF1 rearrangements and EGFR amplification, and a ROS1–EPHA7 fusion. Notably, the ROS1–EPHA7 mutation is a novel fusion, and this is the first time it has been identified in breast cancer tumours. PALB2, STK11 and FGFR2 were detected in only one patient in the very-early-relapsed group. Among those CRGAs, 11 of 23 patients (47.8%) were detected with single actionable alterations. A proportion of 39.1% of patients (*n* = 9) exhibited multiple actionable alterations (Table 2). The patient with the most actionable alterations had four actionable alterations, including BRCA loss, CCND1 amplification, PI3KCA base substitutions and the novel ROS1–EPHA7 fusion that was identified in breast cancer tumours for the first time.

**Table 2.** Actionable CRGAs and on-label and off-label targeted therapies in very early relapsed TNBC patients.

ID	CRGAs	On-label	Off-label
00624	PTEN	Everolimus	Temsirolimus
	BRCA1	Olaparib, Talazoparib	Niraparib, rucaparib
0625	BRCA1	Olaparib, talazoparib	Niraparib, rucaparib
00628	KRAS	NA	Binimetinib, cobimetinib, trametinib
00630	BRCA1	Olaparib, talazoparib	Niraparib, rucaparib
00632	PTEN	Everolimus	Temsirolimus
00634	PTEN	Everolimus	Temsirolimus
	PIK3CA	Everolimus	Temsirolimus
00637	ERBB2	Ado-trastuzumab, emtansine, pertuzumab, trastuzumab, trastuzumab-dkst, trastuzumab-pkrb	Afatinib, dacomitinib
00638	PIK3CA	Everolimus	Temsirolimus
	NF1	NA	Binimetinib, cobimetinib, trametinib
00639	FGFR2	NA	Pazopanib, ponatinib
	PALB2	Olaparib, talazoparib	Niraparib, rucaparib
00641	PIK3CA	Everolimus	Temsirolimus
	STK11	Everolimus	Temsirolimus
00642	PIK3CA	Everolimus	Temsirolimus
	PTEN	Everolimus	Temsirolimus
	NF1	NA	Binimetinib, cobimetinib, trametinib
00643	ERBB2	Ado-trastuzumab, emtansine, pertuzumab, trastuzumab, trastuzumab-dkst, trastuzumab-pkrb	Afatinib, dacomitinib
	PIK3CA	Everolimus	Temsirolimus
	EGFR	Lapatinib	Afatinib, cetuximab, panitumumab, dacomitinib, erlotinib, gefitinib, osimertinib
00645	PTEN	Everolimus	Temsirolimus
	KRAS	NA	Binimetinib, cobimetinib, trametinib
00647	BRCA1	Olaparib, talazoparib	Niraparib, rucaparib
00648	CCND1	Abemaciclib, palbociclib, ribociclib	NA
	PIK3CA	Everolimus	Temsirolimus
	ROS1	NA	Ceritinib, crizotinib, lorlatinib
	BRCA1	Olaparib, talazoparib	Niraparib, rucaparib
00649	PTEN	Everolimus	Temsirolimus
00652	PTEN	Everolimus	Temsirolimus
00653	PTEN	Everolimus	Temsirolimus
00654	CCND1	Abemaciclib, palbociclib, ribociclib	NA
00656	CCND1	Abemaciclib, palbociclib, ribociclib	NA

### Pathway analysis

**Figure 3.**
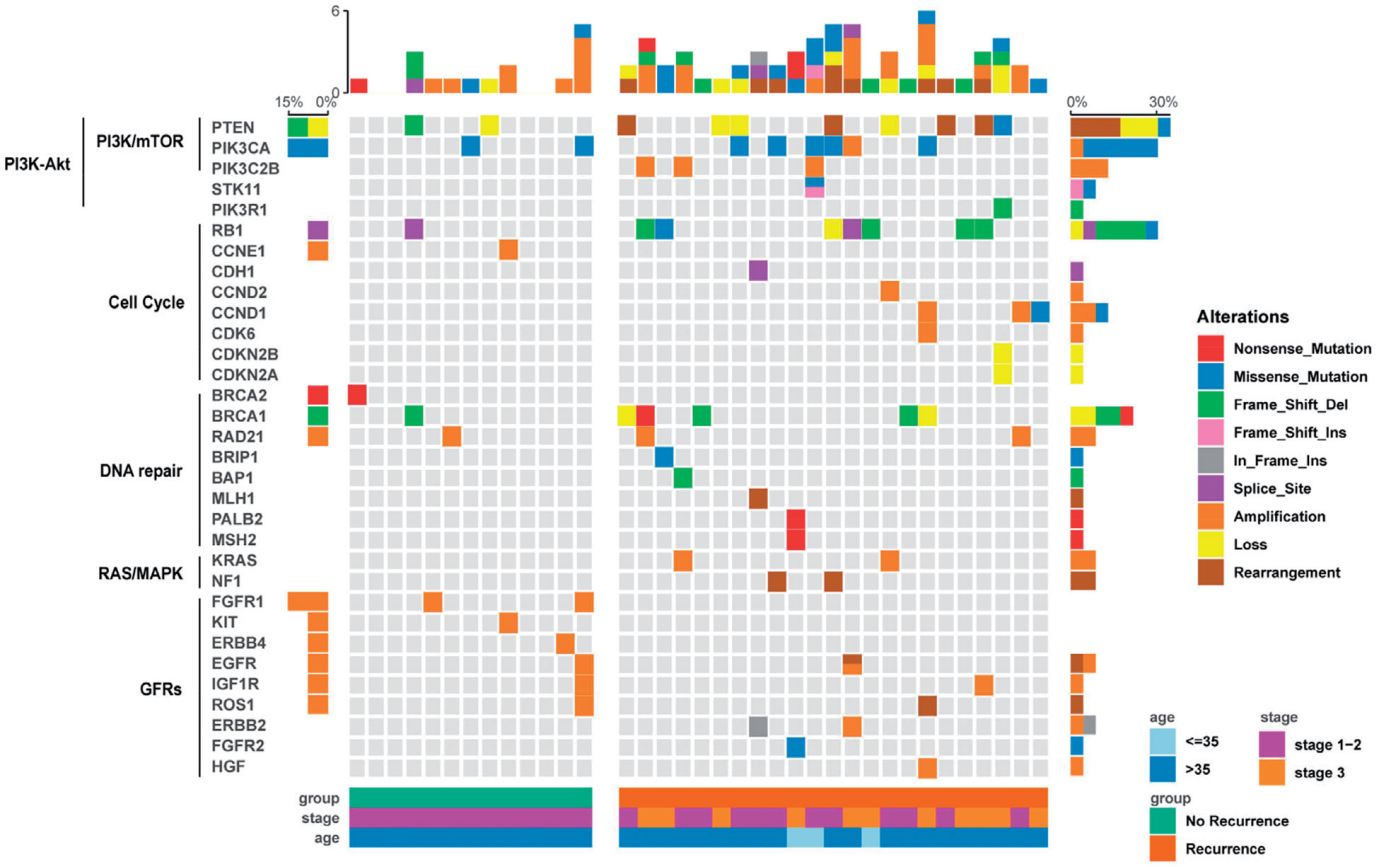
Representation of genomic alterations (GAs) into five functional and targetable pathways.

We explored whether GRGAs in different genes could be clustered in some known pathways. We depicted a pathway mutation status and found the association with the clinical variables. In the very-early-relapsed TNBC group, 61%, 52%, 43%, 22% and 17% of the very early relapsed TNBC patients in our cohort had at least one CRGAs in PI3K/mTOR, cell cycle, DNA repair, growth factor receptors (GFRs) and RAS/MAPK signalling pathways, respectively (Figure 3; Table 3). About 61% cases had identified alterations in PI3K/mTOR pathway including PTEN (35%), PIK3CA (26%), PIK3C2B (13%) and STK11 (4%). For the cell cycle pathway, the most frequent GAs involved were RB1 (30.4%) and CCND1 (13%). In addition, CCND2, CDH1, CDK12, CDK6, CDKN2A and CDKN2B were each found in only one case. The mutation frequency in the DNA repair pathway, GFR pathway and the RAS/MAPK pathway was each depicted (Table 3). Interestingly, the mutation distribution in different signal pathways has a certain tendency. Gene mutations in the same signalling pathway were generally mutually exclusive (Figure 3). In addition, the enrichment of mutations in different signalling pathways was associated with the initial tumour stage. For instance, stage III patients had more PI3K/mTOR and cell cycle pathway mutations; meanwhile, DNA repair pathway mutations and RAS/MAPK signalling pathway mutations are more likely to be detected in stage I and II patients (Figure 3). When comparing stage I–II recurrence-free patients with stage I–II very-early-relapsed patients, no statistical difference was found in PI3K/mTOR (*p*= .115), cell cycle (*p*= .645), DNA repair (*p*= .411) and GFRs (*p*=.322) pathways enrichment. In the no-recurrence group, the percentage of patients who had at least one CRGA in PI3K/mTOR, cell cycle, DNA repair and GFR signalling pathways were 38.5%,15.4%, 38.5% and 23.1%, respectively; no alterations were found in RAS/MAPK signalling pathway. Interestingly, very-early-relapsed TNBCs had more alterations in the cell cycle pathway than the control group, which was statistically significant (Table 3).

**Table 3.** The most prevalent genomic alterations in pathway analysis.

Pathway	Mutation frequency (%)		*p* Value
	Early recurrence TNBCs (*N* = 23)	No recurrence TNBCs (*N* = 13)	
PI3K/mTOR	14 (60.8%)	4 (30.8%)	.164
PTEN	8 (34.7%)	2 (15.4%)	
PIK3CA	6 (26.0%)	2 (15.4%)	
PIK3C2B	3 (13.0%)	0	
PIK3R1	1 (4.3%)	0	
STK11	1 (4.3%)	0	
Cell cycle	12 (52.1%)	2 (15.4%)	.039
RB1	7 (30.4%)	1 (7.7%)	
CCND1	3 (13.0%)	0	
CCNE1	0	1 (7.7%)	
CDK12	1 (4.3%)	0	
CCND2	1 (4.3%)	0	
CDK6	1 (4.3%)	0	
CDKN2B	1 (4.3%)	0	
CDKN2A	1 (4.3%)	0	
DNA repair	10 (43.4%)	3 (23.1%)	.292
BRCA1	5 (21.7%)	1 (7.7%)	
RAD21	2 (8.7%)	1 (7.7%)	
BRCA2	0	1 (7.7%)	
PALB2	1 (4.3%)	0	
MSH1	1 (4.3%)	0	
MLH1	1 (4.3%)	0	
BRIP1	1 (4.3%)	0	
BAP1	1 (4.3%)	0	
GFRs	5 (21.7%)	4 (30.8%)	.693
ERBB2	2 (8.7%)	0	
FGFR1	0	2 (15.4%)	
EGFR	1 (4.3%)	1 (7.7%)	
IGF1R	1 (4.3%)	1 (7.7%)	
FGFR2	1 (4.3%)	0	
ROS1	1 (4.3%)	1 (7.7%)	
ERBB4	0	1 (7.7%)	
KIT	0	1 (7.7%)	
RAS/MAPK	4 (17.3%)	0 (0%)	NS
KRAS	2 (8.7%)	0	
NF1	2 (8.7%)	0	

### Rare ROS1 fusion in breast cancer

**Figure 4.**
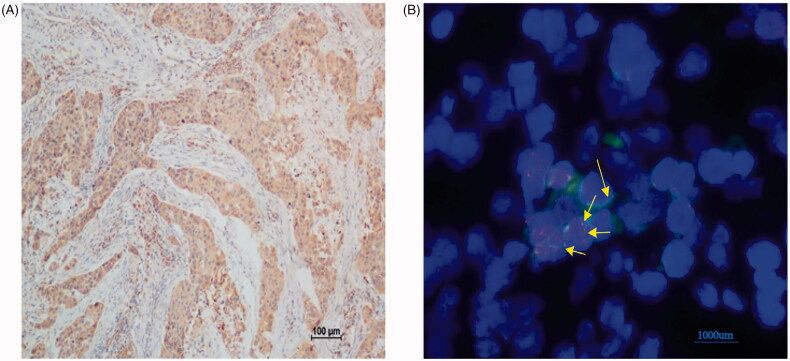
Immunohistochemistry text of ROS1 and fluorescence *in situ* hybridization (FISH) test of ROS1 fusion.

Comprehensive genomic profiling analysis revealed a novel *ROS1–EPHA7* rearrangement. It was found in a 60-year-old patient who was diagnosed with stage IIIC TNBC in September 2016, and the tumour recurred 9 months after surgery. This novel *ROS1–EPHA7* fusion variant is generated by the fusion of introns 1–33 of *ROS1* on chromosome 6q22 to extron 6–17 of *EPHA7* on chromosome 6q16. We performed IHC and found that *ROS1* was diffusely positive in the tumour (Figure 4(A)). The sequencing result was further verified by FISH using a *ROS1* BAP set that showed the presence of a *ROS1* rearrangement with the intact red-fused signal, indicating a ROS1 rearrangement (Figure 4(B)).

## Discussion

TNBC is an aggressive subtype of breast cancer that is characterized by resistance to therapy and poor patient survival. Currently, due to the lack of direct targets for treatment, it is especially important to identify gene mutations that can be used as therapeutic targets in patients with TNBC. To improve patient outcomes and to optimize treatment regimens, novel therapeutic targets need to be identified.

In the present study, using the NGS technique, we aimed to identify novel gene mutations in a cohort of 23 very-early-relapsed TNBC patients to identify new direct targets for treatments. Both the NGS platform and the cancer panel genes chosen in this study were previously used in several other studies on breast cancer [11] and other types of cancers [12].

We identified 137 CRGAs in 23 very-early-relapsed TNBCs, among which TP53, PTEN, RB1, PIK3CA, BRCA1, NOTCH1, MYC and CCND1 were the most frequently mutated genes (Figure 1) in our cohort. Compared with previous studies in TNBC, TP53 was still the most frequently mutated gene, while the mutation frequency of PTEN and RB1 was higher than that reported in other literature [13,14]. It has been reported that those three tumour suppressors are also the most frequent drivers of metastasis in diverse types of solid human cancers, not just in breast cancer [15]. Notably, our study found that seven very-early-relapsed TNBC patients who were detected with RB1 mutations, including four frameshifts and one for each of missense mutation, loss and splice site, had shorter DFS than patients without RB1 mutations; this difference was statistically significant. Compared to a previous study in a large cohort of Chinese TNBC [16], similar cancer-related variations observed in all patients we studied were TP53 mutations, followed by PIK3CA and PTEN mutations. Thus, understanding the impact of these tumour suppressors on clinical outcomes could be valuable.

### ERBB2 mutation

Of further note, two with basal-like subtype patients were detected with ERBB2 mutation (Figure 1(A)), while a previous large cohort of Chinese TNBC showed that five luminal androgen receptor (LAR) patients harbour ERBB2 mutations [16]. One patient detected with ERBB2 amplification was diagnosed with IIIC TNBC in May 2016, and lung metastasis occurred 5 months after surgery. ERBB2 amplification implied that the anti-Her2 theory might be correct. Another patient with ERBB2 IHC (1+) was found with an ERBB2-P780_Y781insGSP mutation, the third most common HER2 exon 20 insertions in lung cancer [17], which indicated that anti-Her2 therapies such as neratinib and trastuzumab ado-trastuzumab emtansine (T-DM1) might benefit patients. Notably, this insertion mutant is located in the Pkinase-Tyr sequence of ERBB2. Mutations in the ERBB2 kinase domain have been identified in about 2–5% of various human cancers [18]. Lapatinib, which is known as a small molecule tyrosine kinase inhibitor (TKI), targeted the kinase domain of ERBB2-approved for breast cancer patients and may be resistant because of this insertion mutant [17]. Another ERBB2 T798I mutation that occurs in the same kinase domain has been demonstrated to cause a strong lapatinib-resistance effect by *in vitro* study [19]. However, whether the ERBB2 kinase domain mutation detected in our study could lead to clinical drug resistance or not has been validated by preclinical studies.

### Rare ROS1 fusion in breast cancer

Comprehensive genomic profiling analysis revealed a novel ROS1–EPHA7 rearrangement. ROS1 is a proto-oncogene located on the long arm of chromosome 6, which encodes a receptor tyrosine kinase (RTK) and is involved in regulating of cancer cell growth and differentiation [20]. EPH receptor family with 14 distinct RTK constitutes an important class of cell surface proteins. Higher expression level of EPHA7 is correlated with poor prognosis and metastasis in breast cancer [21]. ROS1 fusion was detected in 2.59% of Chinese non-small cell lung cancer (NSCLC) patients [22] but has not been found in TNBC patients before. It has been reported that the objective response rate (ORR) of crizotinib in ROS1 fusion NSCLC patients was 83.3% [23]. This is the first report of a ROS1–EPHA7 fusion identified using F1CDx. Notably, further IHC and FISH testing verified the existence of ROS1–EPHA7 fusion on the RNA and protein level, suggesting that the ROS1 fusion may retain the RTK domain. The patient was diagnosed with stage IIIC TNBC and had 9 months of DFS. It was speculated that ROS1–EPHA7 fusion was characterized by strong aggressive, metastasis and poor prognosis in breast cancer. Unfortunately, the patient experienced disease progression after 5-month vinorelbine–capecitabine-combined chemotherapy as the first-line treatment and was then lost to follow-up. Thus, the response to crizotinib could not be observed in this patient.

We also detected a majority of mutations identified in only one patient (Figure 1), which can be explained by the high heterogeneity of TNBC [24]. These low-frequency mutations also have important clinical implications. For instance, ARID1A and MCL-1 have been related to chemotherapy sensitivity, ARID1A down-regulation has been associated with a poorer response to paclitaxel-based chemotherapy in patients with TNBC [25], and MCL, which is frequently co-amplified with MYC, has been associated with resistance to chemotherapy [26,27] and decreased DFS [28]. For *in vitro* studies, the role of IKBKE, IGF1R, NOTCH3 and MDM4 in tumorigenesis and tumour metastasis have been reported [29–32] and have provided clinicians with potential insights for understanding the biological behaviour of TNBC and exploring treatment strategies for heavily treated patients.

### Pathway analysis

The genes that were of significant interest in our study could be enriched in key signalling pathways, like the PI3K/mTOR pathway, GFRs, cell cycle pathway or DNA repair, and alterations in these genes could be a potential therapeutic target. PI3K/mTOR pathway has the highest mutation frequency. In our study, the mutation of PI3K-AKT signalling pathway included the PI3K catalytic subunits (PIK3CA, PIK3CB), PI3K regulatory subunit (PIK3R1), AKT-independent mTOR pathway activator (STK11) and the loss of PTEN [33]. In a preclinical study, TNBC cell lines of M and LAR subtypes preferentially responded to the dual PI3K/mTOR inhibitor NVP-BEZ235 [34]. The benefit of the pan-PI3K inhibitor BKM120 in metastatic TNBC, both in monotherapy and combination therapy with PARP inhibitors, is undergoing clinical research (NCT01629615; NCT01790932; NCT01623349) [35]. The effectiveness of everolimus (the most studied blocking agent aimed at the mTOR kinase) in both primary and metastatic TNBC was confirmed by clinical trials [36,37]. These promising data demonstrate that PI3K inhibitors or mTOR inhibitors may help select TNBC patients with activating mutations in the PI3K-AKT-mTOR pathway.

RAS/MAPK activity can be aberrantly stimulated via the copy number alterations of KRAS and somatic alterations of NF1 [38]. Preclinical studies have demonstrated that basal type breast cancer cells have an activated RAS-like transcriptional program and are significantly more sensitive to MEK inhibitors compared with luminal and HER-2 amplified lines [39]. Treatment with MEK inhibitor caused the up-regulation of PI3K signalling, and the dual inhibition of both pathways could achieve better anti-tumour effects both *in vitro* and *in vivo* [40]. These studies provide a rational hypothesis for patient selection in clinical trials with the aim to evaluate the clinical effect of MEK and PI3K inhibitors in TNBC. Clinical trials of EGFR-targeted TKIs targeting EGFR amplification in TNBC failed in both TKI monotherapy and in combination with chemotherapy [41,42]. It is still controversial if TNBC patients may respond to EGFR-TKI agents.

TNBCs are a highly proliferative group of tumours enriched for high expression of cell-cycle genes, although they are considered to be resistant to CDK4/6 inhibitors. As a heterogeneous disease, and early preclinical study has shown that the LAR subtype of TNBC was highly sensitive to CDK4/6 inhibition both *in vitro* and *in vivo* in MDA-MB-453 LAR cell line xenografts compared with the basal-like subtype [43]. In our study, two patients with LAR subtype harbour CDKN2A loss (in one case) and CCND1 amplification (in one case) in accordance with previous study [16]. The study also illustrated that target the cell cycle pathway might be effective in selected TNBC patients.

Some studies identified a subgroup of TNBC with a deficiency of DNA repair, mainly due to mutations or methylation of BRCA1/2, and other genes involved in DNA damage repair pathway [13,44]. A clinical trial (NCT00494234) for a poly adenosine diphosphate-ribose polymerase (PARP) inhibitor, olaparib, in patients with BRCA1 or BRCA2 mutations and advanced breast cancer, provided an impressive ORR of 44% [45]. A randomized, phase 3 trial in which olaparib monotherapy was compared with standard therapy in patients with a germline BRCA mutation and human epidermal growth factor receptor type 2 (HER2)-negative metastasis breast cancer, detected a longer progression-free survival (PFS) of 7.0 months in the olaparib group than the 4.2 months (HR = 0.58, 95%CI: 0.43–0.80, *p*<.001), but no statistically significant improvement in OS [46,47]. Given that most BRCA1/2 carriers are attributed to TNBC [48], olaparib could provide a significant benefit among TNBC patients deficient in DNA damage repair. Except for BRCA1/2, many mutations associated with TNBC are mainly distributed in DNA damage repair pathway, including the above-mentioned PALB2, RAD21 and MSH2, along with some other genes that were not detected in our study. Therapies designed for these mutated genes are scarce. It is still unclear whether these mutated genes can be treatment targets or not, but the utility of DNA cross-linking agents in combination with targeted agents has been reported to improve the curative effect for patients with DNA damage repair [31].

Our study also has some limitations. First, as a hospital-based retrospective study, the number of our samples was limited by sample quality and patient follow-up. Second, only two of 23 very-early-relapsed breast cancer patients were still under treatment but not with on-label targeted drugs; as a result, the efficacy of the drug predicted by F1CDx cannot be determined in this study. Meanwhile, the patient with the rare ROS1 fusion was lost to follow-up, so whether crizotinib can benefit TNBC patients with ROS1 fusion was not validated in this study. The last but not least, one critical limitation was the use of F1CDx to study genes relevant to recurrence, especially with a small cohort of patients in this study; another limitation was the pathway analysis as none has passed statistical tests.

## Conclusions

In summary, TNBC is a heterogeneous disease, and few recurrent mutations can be identified. Limited treatment options for the relapsed TNBC patients contribute to unfavourable prognosis. NGS-based comprehensive genomic profiling of DNA from breast cancer FFPE tumour tissue specimens to assess potential therapeutic targets is readily available. Target profiling showed a high frequency of GAs linked to potential treatment options with approved or investigational drugs. NGS results demonstrate distinct clinically testable therapeutic hypotheses for individual patients. This innovative approach can provide access to potentially effective drugs and benefit the greatest number of patients in individualized treatment.

## Supplementary Material

Supplemental Material

## Data Availability

The datasets used and analysed during the current study are available from the corresponding author on reasonable request.

## Abbreviations

**Abbreviations**
TNBC: Triple-negative breast cancer
CRGAs: Clinically relevant genomic alterations
NGS: Next-generation sequencing
CGP: Comprehensive genomic profiling
FFPE: Formalin-fixed paraffin-embedded
TMB: Tumour mutational burden
TKI: Tyrosine kinase inhibitor
DFS: Disease-free survival
OS: Overall survival
ORR: Objective response rate
