# Supplementary material for: Clinical genomic profiling to identify actionable alterations for very early relapsed triple-negative breast cancer patients in the Chinese population
Source: Ann Med. 2021 Aug 16;53(1):1358–69. doi: 10.1080/07853890.2021.1966086 (PMC8381897; doi:10.1080/07853890.2021.1966086)
Supplement: Supplemental Material [file IANN_A_1966086_SM7865.docx]

**Supplementary Table 1. Genes with full coding exonic regions included in FoundationOne CDx™ for the detection of substitutions, insertions and deletions (indels), and copy number alterations (CNAs).**

| *ABL1* | *BRAF* | *CDKN1A* | *EPHA3* | *FGFR4* | *IKZF1* | *MCL1* | *NKX2-1* | *PMS2* | *RNF43* | *TET2* |
| --- | --- | --- | --- | --- | --- | --- | --- | --- | --- | --- |
| *ACVR1B* | *BRCA1* | *CDKN1B* | *EPHB1* | *FH* | *INPP4B* | *MDM2* | *NOTCH1* | *POLD1* | *ROS1* | *TGFBR2* |
| *AKT1* | *BRCA2* | *CDKN2A* | *EPHB4* | *FLCN* | *IRF2* | *MDM4* | *NOTCH2* | *POLE* | *RPTOR* | *TIPARP* |
| *AKT2* | *BRD4* | *CDKN2B* | *ERBB2* | *FLT1* | *IRF4* | *MED12* | *NOTCH3* | *PPARG* | *SDHA* | *TNFAIP3* |
| *AKT3* | *BRIP1* | *CDKN2C* | *ERBB3* | *FLT3* | *IRS2* | *MEF2B* | *NPM1* | *PPP2R1A* | *SDHB* | *TNFRSF* |
| *ALK* | *BTG1* | *CEBPA* | *ERBB4* | *FOXL2* | *JAK1* | *MEN1* | *NRAS* | *PPP2R2A* | *SDHC* | *TP53* |
| *ALOX12B* | *BTG2* | *CHEK1* | *ERCC4* | *FUBP1* | *JAK2* | *MERTK* | *NT5C2* | *PRDM1* | *SDHD* | *TSC1* |
| *AMER1* | *BTK* | *CHEK2* | *ERG* | *GABRA6* | *JAK3* | *MET* | *NTRK1* | *PRKAR1A* | *SETD2* | *TSC2* |
| *APC* | *C11orf30* | *CIC* | *ERRFI1* | *GATA3* | *JUN* | *MITF* | *NTRK2* | *PRKCI* | *SF3B1* | *TYRO3* |
| *AR* | *CALR* | *CREBBP* | *ESR1* | *GATA4* | *KDM5A* | *MKNK1* | *NTRK3* | *PTCH1* | *SGK1* | *U2AF1* |
| *ARAF* | *CARD11* | *CRKL* | *EZH2* | *GATA6* | *KDM5C* | *MLH1* | *P2RY8* | *PTEN* | *SMAD2* | *VEGFA* |
| *ARFRP1* | *CASP8* | *CSF1R* | *FAM46C* | *GID4 (C17orf39)* | *KDM6A* | *MPL* | *PALB2* | *PTPN11* | *SMAD4* | *VHL* |
| *ARID1A* | *CBFB* | *CSF3R* | *FANCA* | *GNA11* | *KDR* | *MRE11A* | *PARK2* | *PTPRO* | *SMARCA4* | *WHSC1* |
| *ASXL1* | *CBL* | *CTCF* | *FANCC* | *GNA13* | *KEAP1* | *MSH2* | *PARP1* | *QKI* | *SMARCB1* | *WHSC1L* |
| *ATM* | *CCND1* | *CTNNA1* | *FANCG* | *GNAQ* | *KEL* | *MSH3* | *PARP2* | *RAC1* | *SMO* | *WT1* |
| *ATR* | *CCND2* | *CTNNB1* | *FANCL* | *GNAS* | *KIT* | *MSH6* | *PARP3* | *RAD21* | *SNCAIP* | *XPO1* |
| *ATRX* | *CCND3* | *CUL3* | *FAS* | *GRM3* | *KLHL6* | *MST1R* | *PAX5* | *RAD51* | *SOCS1* | *XRCC2* |
| *AURKA* | *CCNE1* | *CUL4A* | *FBXW7* | *GSK3B* | *KMT2A (MLL)* | *MTAP* | *PBRM1* | *RAD51B* | *SOX2* | *ZNF217* |
| *AURKB* | *CD22* | *CXCR4* | *FGF10* | *H3F3A* | *KMT2D (MLL2)* | *MTOR* | *PDCD1* | *RAD51C* | *SOX9* | *ZNF703* |
| *AXIN1* | *CD274* | *CYP17A1* | *FGF12* | *HDAC1* | *KRAS* | *MUTYH* | *PDCD1LG2* | *RAD51D* | *SPEN* |  |
| *AXL* | *CD70* | *DAXX* | *FGF14* | *HGF* | *LTK* | *MYC* | *PDGFRA* | *RAD52* | *SPOP* |  |
| *BAP1* | *CD79A* | *DDR1* | *FGF19* | *HNF1A* | *LYN* | *MYCL* | *PDGFRB* | *RAD54L* | *SRC* |  |
| *BARD1* | *CD79B* | *DDR2* | *FGF23* | *HRAS* | *MAF* | *MYCN* | *PDK1* | *RAF1* | *STAG2* |  |
| *BCL2* | *CDC73* | *DIS3* | *FGF3* | *HSD3B1* | *MAP2K1* | *MYD88* | *PIK3C2B* | *RARA* | *STAT3* |  |
| *BCL2L1* | *CDH1* | *DNMT3A* | *FGF4* | *ID3* | *MAP2K2* | *NBN* | *PIK3C2G* | *RB1* | *STK11* |  |
| *BCL2L2* | *CDK12* | *DOT1L* | *FGF6* | *IDH1* | *MAP2K4* | *NF1* | *PIK3CA* | *RBM10* | *SUFU* |  |
| *BCL6* | *CDK4* | *EED* | *FGFR1* | *IDH2* | *MAP3K1* | *NF2* | *PIK3CB* | *REL* | *SYK* |  |
| *BCOR* | *CDK6* | *EGFR* | *FGFR2* | *IGF1R* | *MAP3K13* | *NFE2L2* | *PIK3R1* | *RET* | *TBX3* |  |
| *BCORL1* | *CDK8* | *EP300* | *FGFR3* | *IKBKE* | *MAPK1* | *NFKBIA* | *PIM1* | *RICTOR* | *TEK* |  |

**Supplementary Table 2. Genes with select intronic regions for the detection of gene rearrangements, one with 3’UTR, one gene with a promoter region and one ncRNA gene.**

| *ALK*  *introns 18, 19* | *BRCA1*  *introns 2, 7, 8,*  *12, 16, 19, 20* | *ETV4*  *introns 5, 6* | EZR  *introns 9- 11* | *KIT*  *intron 16* | *MYC*  *intron 1* | *NUTM1*  *intron 1* | *RET*  *introns 7-11* | *SLC34A2*  *intron 4* |
| --- | --- | --- | --- | --- | --- | --- | --- | --- |
| *BCL2 3’UTR* | *BRCA2*  *intron 2* | *ETV5*  *introns 6, 7* | *FGFR1*  *intron 1, 5, 17* | *KMT2A (MLL)*  *introns 6-11* | *NOTCH2*  *intron 26* | *PDGFRA*  *introns 7, 9, 11* | *ROS1*  *introns 31-35* | *TERC*  *ncRNA* |
| *BCR*  *introns 8, 13,*  *14* | *CD74*  *introns 6- 8* | *ETV6*  *introns 5, 6* | *FGFR2*  *intron 1, 17* | *MSH2*  *intron 5* | *NTRK1*  *introns 8-10* | *RAF1*  *introns 4-8* | *RSPO2*  *intron 1* | *TERT*  Promoter |
| *BRAF*  *introns 7- 10* | *EGFR*  *introns 7, 15,*  *24-27* | *EWSR1*  *introns 7-13* | *FGFR3*  *intron 17* | *MYB*  *intron 14* | *NTRK2*  *Intron 12* | *RARA*  *intron 2* | *SDC4*  *intron 2* | *TMPRSS2*  *introns 1- 3* |
